# Safety of Neoadjuvant Immunotherapy in Resectable Cancers: A Meta-Analysis

**DOI:** 10.3389/fimmu.2022.802672

**Published:** 2022-01-31

**Authors:** Jiawei Xu, Yongfeng Wu, Yuedan Xu, Yuan Qiu, Xiaobo Li, Yumeng Song, Ling Zhang

**Affiliations:** ^1^ Stomatology Hospital, School of Stomatology, Zhejiang University School of Medicine, Zhejiang Provincial Clinical Research Center for Oral Diseases, Key Laboratory of Oral Biomedical Research of Zhejiang Province, Cancer Center of Zhejiang University, Hangzhou, China; ^2^ Department of Thoracic Surgery, The First Affiliated Hospital, School of Medicine, Zhejiang University, Hangzhou, China

**Keywords:** neoadjuvant immunotherapy, immune checkpoint inhibitors, safety, treatment-related adverse events, immune-related adverse events, meta-analysis

## Abstract

**Background:**

Neoadjuvant immunotherapy has preliminarily been effective in multiple resectable cancers. However, its safety is still largely unknown.

**Methods:**

A systematic literature search was conducted in PubMed, Embase, Web of Science, and Cochrane Library up to February 28th, 2021. Pooled incidence and risk ratio (RR) of adverse events were calculated using the R software.

**Results:**

Twenty-eight studies involving 2863 patients were included. First, the incidence for all-grade treatment-related adverse events (trAEs) was 94% (95% CI, 81%-98%), with 43% (95% CI, 24%-64%) for high-grade trAEs. For different treatment groups, neoadjuvant immune checkpoint inhibitors (ICIs) plus chemotherapy was associated with a higher incidence of all-grade [99% (95% CI, 98%-99%) *vs.* 76% (95% CI 47%-92%); *P* < 0.001] and high-grade [80% (58%-92%) *vs.* 15% (9%-24%); *P* < 0.001] trAEs compared with neoadjuvant ICIs alone. The most common high-grade trAEs were lipase increased (5%; 95% CI, 2%-10%), colitis (3%; 95% CI, 0-7%) and transaminitis (3%; 95% CI, 0-7%) for neoadjuvant ICIs, and neutropenia (53%; 95% CI, 31%-74%), anemia (8%; 95% CI, 3%-15%) and AST increased (4%; 95% CI, 2%-7%) for neoadjuvant ICIs plus chemotherapy. Furthermore, the incidence rates of progressive disease while on treatment, treatment-related surgical delays and deaths were 6% (95% CI, 4%-10%), 3.2% (12 of 377 patients) and 0.47% (5 of 1075 patients), respectively.

**Conclusion:**

Compared with neoadjuvant ICIs alone, neoadjuvant ICIs plus chemotherapy had a higher incidence of trAEs. In addition, neoadjuvant immunotherapy had a low rate of progressive diseases, surgical delays and deaths.

## Introduction

Immune checkpoint inhibitors (ICIs) have demonstrated remarkable therapeutic efficacy in various advanced malignancies (1). In view of the outstanding efficacy of immunotherapy in advanced cancers, the application of ICIs in earlier stages is further developed to improve curability and survival (2). It seems that ICIs may be more effective when the primary tumor is in place because they can leverage a high level of endogenous tumor antigen to enhance T cell priming (3). Neoadjuvant immunotherapy can also offer additional advantages, such as reducing the tumor burden prior to surgery (2). A recent meta-analysis by Jia et al. (4) reveals that in non-small cell lung cancer (NSCLC), the major pathological response and pathological complete response rates of neoadjuvant ICIs are several times higher than that of neoadjuvant chemotherapy. Due to the significant therapeutic effect, an increasing number of clinical trials explore the efficiency of neoadjuvant immunotherapy.

However, with the start of T cell-mediated antitumor immunity, ICIs could induce the infiltration of immune cells into normal tissues and unleash T cells with subsequent production of proinflammatory cytokines such as interleukin-2 and interleukin-7 (5). The increasing accumulation of immune activation caused by ICIs in normal tissues may be responsible for different kinds of significant autoimmune-mediated adverse events in various organs, including skin, lung, gastrointestinal tract, liver, and the endocrine system, which are called immune-related adverse events (irAEs) (5). Most irAEs tend to be self-limiting or could be ameliorated by several strategies (6). However, in some conditions, life-threatening and fatal events could occur (7). In addition, in neoadjuvant immunotherapy, these adverse events may lead to undue surgical delay and even loss of the opportunity for surgery (2).

Although an increasing number of studies have reported the safety of neoadjuvant immunotherapy, there is still a lack of comprehensive understanding. Here, a meta-analysis was conducted to comprehensively assess the safety of neoadjuvant immunotherapy.

## Methods

### Data Sources, Search Strategy and Selection Criteria

A systematic literature search was conducted in PubMed, EMBASE, Web of Science and Cochrane Library up to February 28th, 2021. The search term was as follows: [neoadjuvant OR “Neoadjuvant Therapy” (Mesh)] AND (“PD-1” OR “PD1” OR “PDCD1” OR “CD279” OR “Programmed Cell Death 1” OR “Programmed Cell Death 1 Receptor” [Mesh] OR “PD-L1” OR “PDL1” OR “CD274” OR “PDCD1L1” OR “Programmed Death Ligand 1” OR “B7-H1 Antigen” [Mesh] OR “CTLA-4” OR “CTLA4” OR “CD152” OR “cytotoxic T-lymphocyte antigen-4” OR “CTLA-4 Antigen” [Mesh] OR “ICI” OR “ICIs” OR “ICB” OR “ICBs” OR “immune checkpoint inhibitor” OR “immune checkpoint inhibitors” OR “immune checkpoint blocker” OR “immune checkpoint blockers” OR “Immune Checkpoint Inhibitors” [Mesh] OR Ipilimumab OR Tremelimumab OR Nivolumab OR Pembrolizumab OR Atezolizumab OR Avelumab OR Durvalumab OR Camrelizumab OR Toripalimab OR Tislelizumab OR Dostarlimab OR Cemiplimab OR Yervoy OR Opdivo OR Keytruda OR Tecentriq OR Bavencio OR Imfinzi OR AiRuiKa OR Jemperli OR Libtayo) AND (cancer OR tumor OR carcinoma OR “Neoplasms” [Mesh] OR “Carcinoma” [Mesh]). Reviews, letters, editorials, comments, meeting abstracts and case reports were not included. Data from different treatment arms within the same study were extracted and reported separately. The references of relevant articles and reviews were also searched for additional eligible studies potentially overlooked.

To be eligible, studies had to satisfy all the following inclusion criteria: (1) studies included cancer patients treated with neoadjuvant ICIs, neoadjuvant ICIs plus adjuvant ICIs, or neoadjuvant ICIs plus chemotherapy; (2) studies clearly reported the incidence of adverse events; (3) studies were published in English. When duplicate reports were identified, the one with a larger sample size and more detailed information was selected.

Two authors (JX and YW) carried out the systematic literature search independently. If there were any disagreements, the study would be re-evaluated by a third investigator (YX).

This meta-analysis was conducted according to the Preferred Reporting Items for Systematic Reviews and Meta-Analyses (PRISMA) guidelines (8).

### Data Extraction and Quality Assessment

The title, first author, publication year, cancer type, drug, dose, phase of the trial, number of participants, and criteria for adverse events reported in each article were extracted. In addition, all-grade and high-grade (grade 3 or higher) adverse events data were also extracted separately. Data extraction was conducted independently by two investigators (JX and YW), and any discrepancies were resolved by discussing with a third author (YX).

The Cochrane Collaboration’s tool was used to evaluate the quality and risk of bias of the included articles (9), including sequence generation, allocation concealment, blinding of participants and personnel, incomplete outcome data, selective outcome reporting, and other sources of bias. Disagreements between investigators were resolved through discussion.

### Statistical Analysis

The primary endpoint of this meta-analysis was to determine the overall incidence of adverse events in neoadjuvant immunotherapy. All-grade and high-grade adverse events were calculated respectively. In addition, subgroup analysis was conducted in different treatment groups to further explore the safety of different treatment modalities based on neoadjuvant immunotherapy. Rate consolidation was conducted using five methods (untransformed, log transformation, logit transformation, arcsine transformation, and Freeman-Tukey double arcsine transformation). The method with results closest to the normal distribution was selected. Statistical heterogeneity in the included studies was assessed using Cochrane’s Q statistic, and I^2^ statistic was used to quantify the inconsistency. The I^2^ cutoffs used to determine inconsistency were very low (< 25%), low (25% to < 50%), moderate (50% to < 75%), and large (> 75%). A fixed-effects model was adopted to pool the results if significant heterogeneity was not present (I^2^ < 50%). Otherwise, a random-effects model was used. Chi-squared test was used to compare the incidence of adverse events between different groups. In addition, the incidence rates of specific types of adverse events were also calculated in different groups. We focused on adverse events reported by at least 10% of the studies.

The secondary objective was to compare the incidence of adverse events between the neoadjuvant ICIs plus chemotherapy group and the chemotherapy control group in the included randomized controlled trials (RCTs). Pooled risk ratio (RR) and 95% confidence intervals (CIs) were calculated. A fixed-effects model or a random-effects model was adopted due to the heterogeneity described above.

In addition, the incidence of treatment-related events, including progressive diseases, surgical delays and deaths, were calculated to further evaluate the safety of neoadjuvant immunotherapy. The incidence rates of surgical delays and deaths were calculated by dividing the total number of surgical delays or deaths by the total number of patients in the relevant studies.

All the analyses above were performed using R software, version 4.0.0 (R Foundation for Statistical Computing) with the package Meta and the function of metaprop. A two-sided *P* < 0.05 was considered statistically significant for all the analyses.

## Results

### Eligible Studies and Characteristics

**Figure 1:**
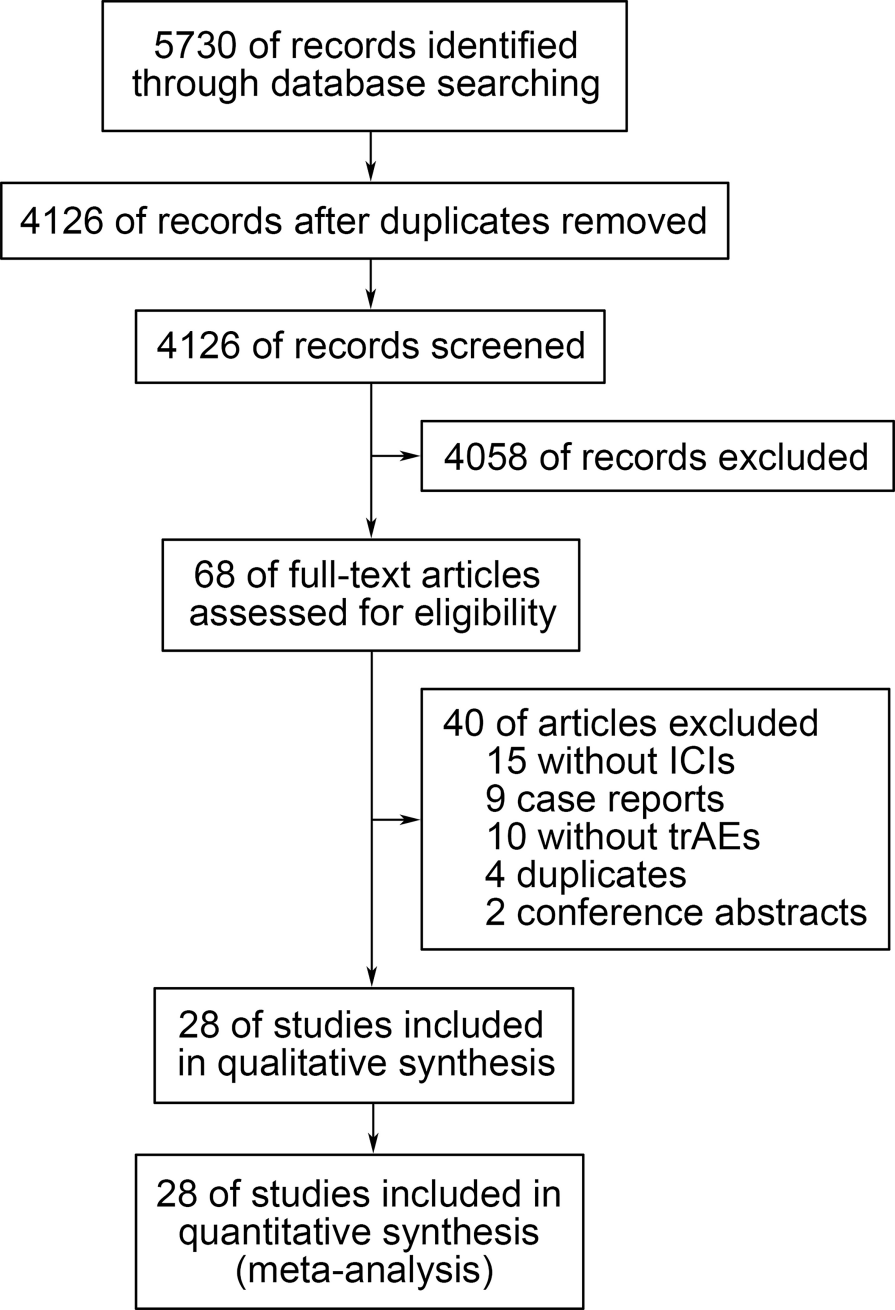
Flow diagram of the study selection process. trAEs, treatment-related adverse events; ICIs, immune checkpoint inhibitors.

The systematic literature search brought up 5730 records, from which 68 potentially eligible studies were collected after screening the titles and abstracts. Ultimately, 28 studies were selected after reviewing the full text (10–37). The reasons for exclusion were as follows: 15 studies did not include ICIs, 9 studies were case reports, 10 studies did not include adverse events data, 4 studies were duplicate reports, and 2 studies were conference abstracts. The detailed retrieval process was shown in **Figure 1**.

The characteristics of the 28 included studies were summarized in **Table S1**. In brief, there were 2863 participants from 25 countries, including 4 RCTs (20–22, 29) comparing the adverse events between neoadjuvant immunotherapy plus chemotherapy and neoadjuvant chemotherapy alone. In terms of the types of treatment, there were 16 studies involving neoadjuvant ICIs only (10, 12, 13, 15–19, 23, 24, 26–28, 31, 34, 36), 8 studies involving neoadjuvant ICIs plus chemotherapy (20–22, 25, 29, 30, 32, 37), and 4 studies involving neoadjuvant ICIs plus adjuvant ICIs (11, 14, 33, 35). Concerning cancer types, the studies included patients with melanoma (n = 6), lung cancer (n = 6), glioblastoma (n = 2), breast cancer (n = 5), bladder cancer (n = 4), Merkel cell carcinoma (n = 1), oropharynx cancer (n = 2), head and neck cancer (n = 1), and colon cancer (n = 1). In addition, the evaluation of adverse events in these studies was mostly based on the Common Terminology Criteria for Adverse Events (CTCAE) version 3.0, 4.0 or 5.0.

### Overall Incidence of Treatment-Related Adverse Events (trAEs)

Among the studies included, the overall incidence of trAEs was available in 16 studies. The overall incidence of all-grade trAEs was 94% (95% CI, 81%-98%; **Figure S1**). Concerning different treatment groups (**Table 1**), the overall incidence of all-grade trAEs was 76% (95% CI, 47%-92%) for the neoadjuvant ICIs group, 99% (95% CI, 98%-99%) for the neoadjuvant ICIs plus chemotherapy group, and 68% (95% CI, 12%-97%) for the neoadjuvant ICIs plus adjuvant ICIs group. Neoadjuvant ICIs plus chemotherapy was associated with a higher overall incidence of all-grade trAEs compared with neoadjuvant ICIs (*P* < 0.001). However, there was no significant difference between neoadjuvant ICIs plus adjuvant ICIs and neoadjuvant ICIs (*P* = 0.780).

**Table 1:** Comparison of the incidence of treatment-related adverse events in different treatment groups.

Type	Treatment Group	Proportion (95% CI) (%)	*P*
All-grade trAEs	Neoadjuvant ICIs	76 (47-92)	/
	Neoadjuvant ICIs plus chemotherapy	99 (98-99)	<0.001^†^
	Neoadjuvant ICIs plus adjuvant ICIs	68 (12-97)	0.780^†^
High-grade trAEs	Neoadjuvant ICIs	15 (9-24)	/
	Neoadjuvant ICIs plus chemotherapy	80 (58-92)	< 0.001^†^
	Neoadjuvant ICIs plus adjuvant ICIs	32 (5-79)	0.377^†^

^†^Compared with neoadjuvant ICIs group.

trAEs, treatment-related adverse events; CI, confidence interval; ICIs, immune checkpoint inhibitors.

In addition, the overall incidence of high-grade trAEs was 43% (95% CI, 24%-64%; **Figure S2**). Concerning different treatment groups (**Table 1**), the overall incidence of high-grade trAEs was 15% (95% CI, 9%-24%) for the neoadjuvant ICIs group, 80% (95% CI, 58%-92%) for the neoadjuvant ICIs plus chemotherapy group, and 32% (95% CI, 5%-79%) for the neoadjuvant ICIs plus adjuvant ICIs group. Similarly, neoadjuvant ICIs plus chemotherapy was associated with a higher overall incidence of high-grade trAEs compared with neoadjuvant ICIs (*P* < 0.001). However, there was no significant difference between neoadjuvant ICIs plus adjuvant ICIs and neoadjuvant ICIs (*P* = 0.377).

### Comparison of the Incidence of trAEs in Different Groups

We compared the incidence of trAEs between the combined ICIs group and the single ICI group to further explore the influence of combined ICIs on trAEs in neoadjuvant immunotherapy. As shown in **Table S2**, the overall incidence of all-grade trAEs was 84% (95% CI, 73%-94%) for combined ICIs and 60% (95% CI, 38%-82%) for single ICI. In addition, the overall incidence of high-grade trAEs was 24% (95% CI, 10%-48%) for combined ICIs and 10% (95% CI, 7%-15%) for single ICI. Although combined ICIs appeared to have a higher incidence of trAEs than single ICI, the statistical difference was not significant (for all-grade: *P* = 0.057; for high-grade: *P* = 0.148).

To determine the impact of the number of cycles on the incidence of trAEs, the incidence of trAEs was compared between different ICI cycles. As shown in **Table S3**, in neoadjuvant ICIs, the incidences of all-grade trAEs [81% (95% CI, 69%-94%) *vs.* 61% (95% CI, 33%-89%), *P* = 0.003] were higher in ≥ 3 cycles than in < 3 cycles, with no difference in high-grade trAEs between the two groups. In addition, in neoadjuvant ICIs plus chemotherapy, the incidence of high-grade trAEs was higher in ≥ 4 cycles than in < 4 cycles [90% (95% CI, 80%-99%) *vs.* 47% (95% CI, 33%-61%), *P* < 0.001].

To compare the incidence of trAEs in immunotherapy between the neoadjuvant group and the advanced group, the incidence of trAEs in advanced cancer immunotherapy was obtained in two meta-analyses (38, 39), and the incidence of trAEs was recalculated using our method. As shown in **Table S4**, the recalculated incidence for all-grade trAEs was 71% (95% CI, 68%-74%) in neoadjuvant ICIs, and 98% (95% CI, 97%-99%) in neoadjuvant ICIs plus chemotherapy. Concerning high-grade trAEs, the incidence was 15% (95% CI, 13%-16%) in neoadjuvant ICIs and 67% (95% CI, 62%-72%) in neoadjuvant ICIs plus chemotherapy. There were no significant differences between the neoadjuvant and advanced groups, neither for all-grade trAEs nor high-grade trAEs.

### Overview of Types of Adverse Events

**Figure 2:**
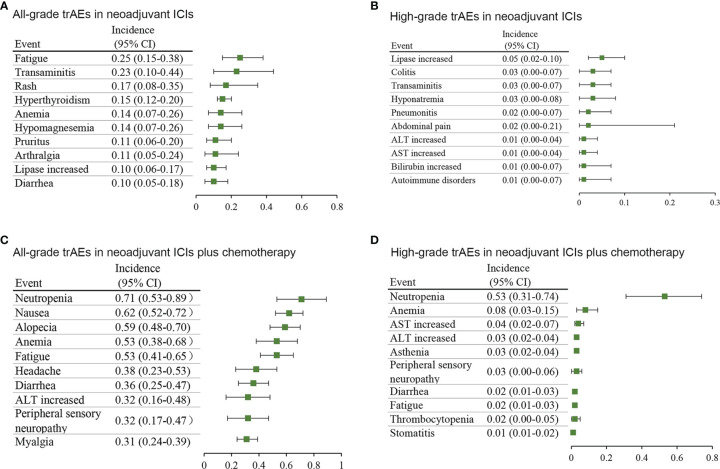
Incidence of the most common treatment-related adverse events in different treatment groups. **(A)** all-grade and **(B)** high-grade in neoadjuvant ICIs alone. **(C)** all-grade and **(D)** high-grade in neoadjuvant ICIs plus chemotherapy. AST, aspartate aminotransferase; ALT, alanine transaminase; CI, confidence interval.

To further discover the distribution of adverse events, we focused on trAEs and irAEs which were reported by at least 10% of the included studies. As shown in **Tables S5–S8**, 51 trAE types and 24 irAE types from the neoadjuvant ICIs group and 55 trAE types and 12 irAE types from the neoadjuvant ICIs plus chemotherapy group were selected for further analyses. Concerning neoadjuvant ICIs, the most common all-grade trAEs were fatigue (25%; 95% CI, 15%-38%), transaminitis (23%; 95% CI, 10%-44%), and rash (17%; 95% CI, 8%-35%) (**Figure 2A**), and the most common high-grade trAEs were lipase increased (5%; 95% CI, 2%-10%), colitis (3%; 95% CI, 0-7%), and transaminitis (3%; 95% CI, 0-7%) (**Figure 2B**). Concerning neoadjuvant ICIs plus chemotherapy, the most common all-grade trAEs were neutropenia (71%; 95% CI, 53%-89%), nausea (62%; 95% CI, 52%-72%), and alopecia (59%; 95% CI, 48%-70%) (**Figure 2C**), and the most common high-grade trAEs were neutropenia (53%; 95% CI, 31%-74%), anemia (8%; 95% CI, 3%-15%), and aspartate aminotransferase (AST) increased (4%; 95% CI, 2%-7%) (**Figure 2D**).

**Figure 3:**
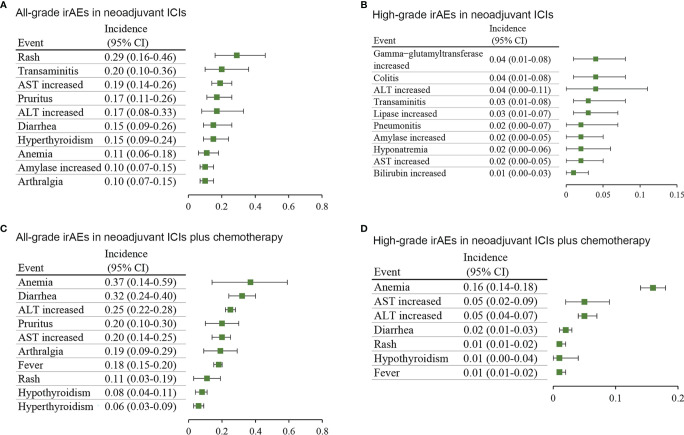
Incidence of the most common immune-related adverse events in different treatment groups. **(A)** all-grade and **(B)** high-grade in neoadjuvant ICIs alone. **(C)** all-grade and **(D)** high-grade in neoadjuvant ICIs plus chemotherapy. AST, aspartate aminotransferase; ALT, alanine transaminase; CI, confidence interval.

In addition, concerning neoadjuvant ICIs, the most common all-grade irAEs were rash (29%; 95% CI, 16%-46%), transaminitis (20%; 95% CI, 10%-36%), and AST increased (19%; 95% CI, 14%-26%) (**Figure 3A**), and the most common high-grade irAEs were gamma-glutamyltransferase increased (4%; 95% CI, 1%-8%), colitis (4%; 95% CI, 1%-8%), and alanine transaminase (ALT) increased (4%; 95% CI, 0-11%) (**Figure 3B**). Concerning neoadjuvant ICIs plus chemotherapy, the most common all-grade irAEs were anemia (37%; 95% CI, 14%-59%), diarrhea (32%; 95% CI, 24%-40%), and ALT increased (25%; 95% CI, 22%-28%) (**Figure 3C**), and the most common high-grade irAEs were anemia (16%; 95% CI, 14%-18%), AST increased (5%; 95% CI, 2%-9%), and ALT increased (5%; 95% CI, 4%-7%) (**Figure 3D**).

**Figure 4:**
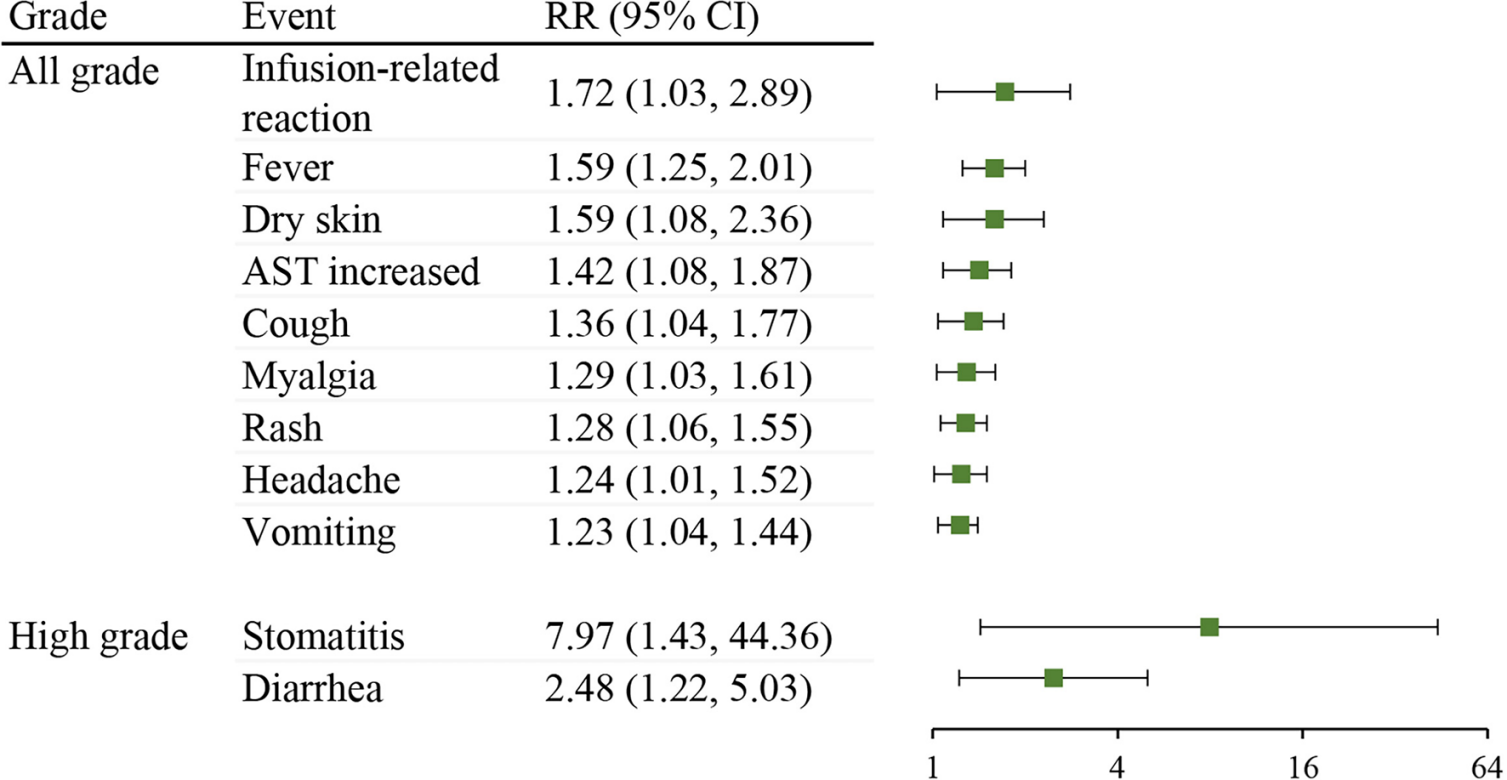
Comparison of treatment-related adverse event types between neoadjuvant ICIs plus chemotherapy with neoadjuvant chemotherapy control arm in RCTs. RR, risk ratio; CI, confidence interval; AST, aspartate aminotransferase.

In addition, to further explore the specific trAE types in neoadjuvant immunotherapy, a comparison was made between neoadjuvant ICIs plus chemotherapy and neoadjuvant chemotherapy control arm in RCTs. As shown in **Figure 4**, compared with the chemotherapy control group, the neoadjuvant ICIs plus chemotherapy group was at a higher risk of all-grade infusion-related reaction (RR 1.72; 95% CI, 1.03-2.89), fever (RR 1.59; 95% CI, 1.25-2.01), dry skin (RR 1.59; 95% CI, 1.08-2.36), AST increased (RR 1.42; 95% CI, 1.08-1.87), cough (RR 1.36; 95% CI, 1.04-1.77), myalgia (RR 1.29; 95% CI, 1.03-1.61), rash (RR 1.28; 95% CI, 1.06-1.55), headache (RR 1.24; 95% CI, 1.01-1.52), vomiting (RR 1.23; 95% CI, 1.04-1.44), high-grade stomatitis (RR 7.97; 95% CI, 1.43-44.36), and diarrhea (RR 2.48; 95% CI, 1.22-5.03).

### Pooled Analysis of Treatment-Related Events in Neoadjuvant Immunotherapy

**Figure 5:**
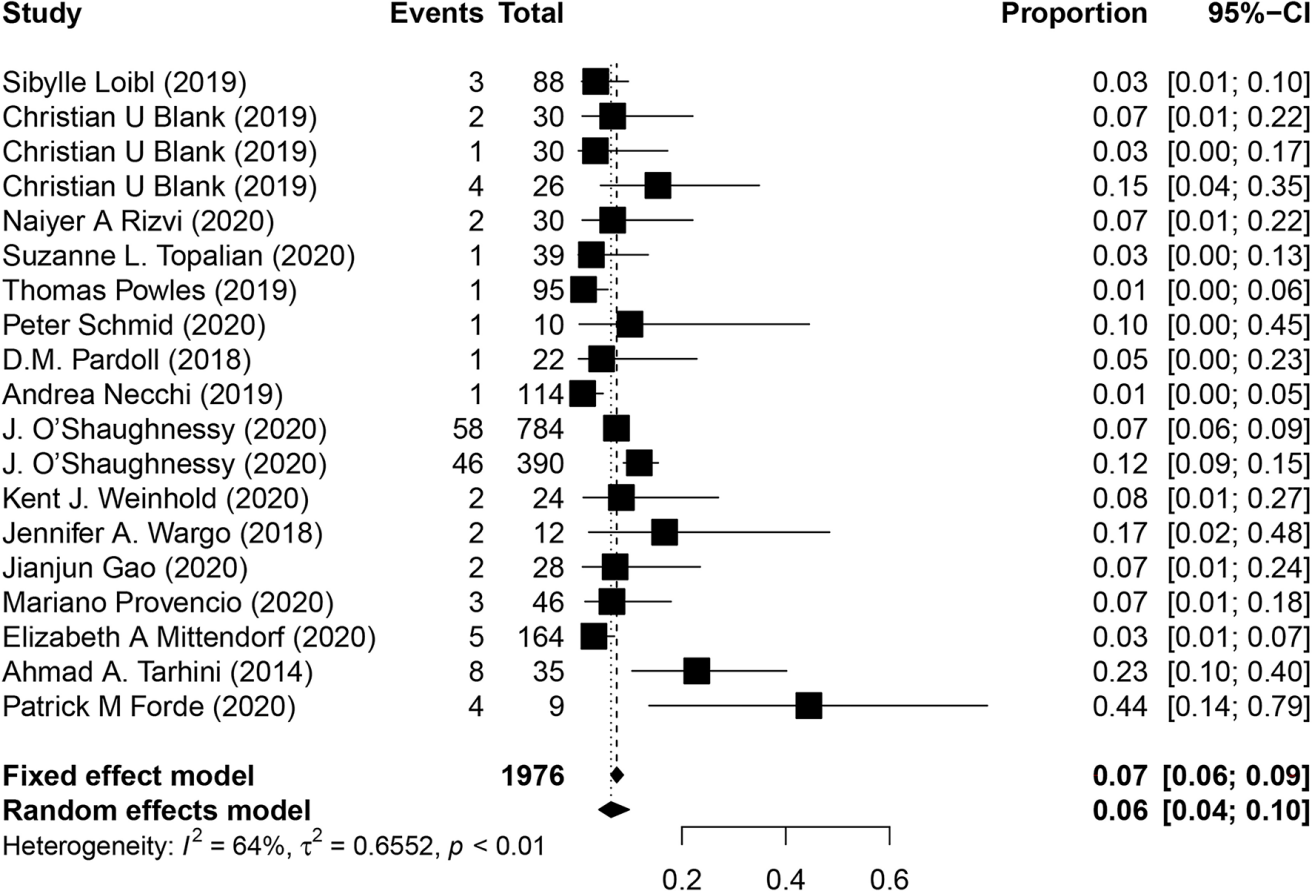
Pooled analysis of progressive diseases in neoadjuvant immunotherapy. CI, confidence interval.

The incidence of progressive diseases while on treatment, treatment-related surgical delays, and deaths are essential for the evaluation of safety in neoadjuvant immunotherapy. Sixteen studies reported the rate of progressive diseases, and the overall incidence was 6% (95% CI, 4%-10%; **Figure 5**). Data for surgical delays was available from 13 studies, and the overall incidence of treatment-related surgical delays was 3.2% (12 of 377). In addition, 13 studies evaluated whether any treatment-related deaths occurred, of which, only 3 studies reported at least one treatment-related death, and the overall incidence was 0.47% (5 of 1075 patients).

### Quality Assessment

Since most of the studies were not blinded, investigators knew which patients had received which treatment and the possible side effects. Therefore, adverse events might have been over-reported. Most information was retrieved from trials with a moderate risk of bias. Further details regarding the quality assessment are available in **Table S9**.

## Discussion

Although the efficacy of neoadjuvant immunotherapy has been preliminarily confirmed, its safety remains controversial. In this study, we comprehensively analyzed the safety of neoadjuvant immunotherapy. First, we compared the incidence of trAEs in different treatment groups, and found that the incidence of trAEs in the neoadjuvant ICIs plus chemotherapy group might be higher than that in the neoadjuvant ICIs group. The findings should be considered from two aspects. First, the high incidence in neoadjuvant ICIs plus chemotherapy deserves attention, and its mechanism and solution strategy require further research. On the other hand, however, it has been reported that the incidence of adverse events could predict the efficacy of ICIs (40). Therefore, whether the high incidence of trAEs is related to its efficacy should be further validated in neoadjuvant immunotherapy plus chemotherapy. Second, although the statistical difference was not significant, there was a trend that the incidence of trAEs of combined immunotherapy seemed to be higher than that of immune monotherapy, requiring confirmation in further trials, and the phenomena should also be considered from two aspects, as mentioned above. In addition, although not present in all comparisons, more immunotherapy cycles were correlated with a higher incidence of trAEs in neoadjuvant ICIs with or without chemotherapy. Since neoadjuvant immunotherapy has emerged recently, the relationship between the number of treatment cycles and the efficacy is still unclear. According to our findings, the balance between the number of cycles and adverse events should also be considered in the future.

In addition, different types of trAEs and irAEs were identified in the neoadjuvant ICIs and neoadjuvant ICIs plus chemotherapy groups. In the neoadjuvant ICIs group, digestive and hepatic-related adverse events were largely identified, including transaminitis (ALT, AST, and gamma−glutamyltransferase), bilirubin, lipase, amylase increased, diarrhea, and colitis, which was similar to adverse events in advanced stage (39). Besides, adverse events of immunotherapy in neoadjuvant and advanced stages both had some high-incidence adverse events such as fatigue, rash, and pruritus. In addition, thyroid-related adverse events (hyperthyroidism and hypothyroidism) were the most common endocrine dysfunctions in the two groups. Concerning neoadjuvant ICIs plus chemotherapy, hematology-related adverse events (neutropenia and anemia) had the highest incidence, consistent with ICIs plus chemotherapy in advanced cancers (38), and the phenomenon is mainly due to the cytological toxicity of chemotherapy.

Moreover, treatment-related specific events, including progressive diseases, surgical delays and deaths, are of great concern to doctors in neoadjuvant immunotherapy. The pooled incidence of progressive diseases was about 6%, considered relatively low. More importantly, in most cases, patients with progressive diseases during neoadjuvant immunotherapy could still be cured through timely surgery. Another interesting aspect is that neoadjuvant immunotherapy might be able to explore the mechanisms of pseudoprogression and hyperprogression reported before (41) due to the ease of obtaining more tissue samples. In addition, treatment-related surgical delays (3.2%) and deaths (0.47%) were low, further confirming the safety and feasibility of neoadjuvant immunotherapy.

The present study had several strengths. A major strength of this study was that we analyzed the safety of neoadjuvant immunotherapy from several aspects, including the overall incidence of trAEs, specific types of trAEs and irAEs, and several treatment-related events. In addition, various comparisons further clarified the pattern of adverse events in neoadjuvant immunotherapy.

However, this study had several limitations. Firstly, the sample sizes of included studies varied significantly, which might explain the significant heterogeneity in some results. Secondly, the current number of studies was still insufficient to analyze safety in specific subgroups such as different cancers and anti-PD-(L)1 drugs. Therefore, further large-scale studies are required in the future, especially RCTs. In addition, some studies report the safety incompletely, especially the incidence of irAEs, which is important to understand the immunotoxicity in neoadjuvant immunotherapy. Furthermore, as treatment-related specific events such as surgical delays and deaths are not detailly reported in RCTs, the difference of safety between neoadjuvant immunotherapy and neoadjuvant chemotherapy is still largely unknown. We hope that the safety data will be reported more completely in future research, which is important in guiding clinical treatment and management.

To the best of our knowledge, it is the first study to analyze the safety of neoadjuvant immunotherapy in multiple malignancies comprehensively. Several conclusions were reached in the study. First, compared with neoadjuvant ICIs alone, neoadjuvant ICIs plus chemotherapy resulted a higher incidence of trAEs. In addition, increased ICI cycles tended to have a higher incidence of trAEs. Furthermore, neoadjuvant immunotherapy had a low rate of progressive diseases, surgery delays, and deaths.

## Data Availability Statement

The original contributions presented in the study are included in the article/**Supplementary Material**. Further inquiries can be directed to the corresponding author.

## Author Contributions

LZ, JX, and YW designed the study. JX, YW, and YX performed the literature search, data extraction and quality assessment. YX and YQ performed statistical analysis, and XL and YS provided assistance. JX drafted the manuscript, and LZ revised the manuscript and had the right to grant on behalf of all authors. All the authors read and approved the final manuscript.

## Conflict of Interest

The authors declare that the research was conducted in the absence of any commercial or financial relationships that could be construed as a potential conflict of interest.

## Publisher’s Note

All claims expressed in this article are solely those of the authors and do not necessarily represent those of their affiliated organizations, or those of the publisher, the editors and the reviewers. Any product that may be evaluated in this article, or claim that may be made by its manufacturer, is not guaranteed or endorsed by the publisher.

## Abbreviations

ICIs, immune checkpoint inhibitors; RR, risk ratio; CI, confidence interval; trAEs, treatment-related adverse events; irAEs, immune-related adverse events; CTCAE, Common Terminology Criteria for Adverse Events; RCTs, randomized controlled trials; PRISMA, Preferred Reporting Items for Systematic Reviews and Meta-Analyses; AST, aspartate aminotransferase; ALT, alanine transaminase.
