# Supplementary material for: Safety of Neoadjuvant Immunotherapy in Resectable Cancers: A Meta-Analysis
Source: Front Immunol. 2022 Jan 31;13:802672. doi: 10.3389/fimmu.2022.802672 (PMC8841351; doi:10.3389/fimmu.2022.802672)
Supplement: Supplementary file 1 [file DataSheet_1.pdf]

Safety of neoadjuvant immunotherapy in resectable cancers: a meta-analysis

Supplementary material

Table S1. Characteristics of the included studies

Figure S1. Forest plots of the incidence of all-grade treatment-related adverse events

Figure S2. Forest plots of the incidence of high-grade treatment-related adverse events

Table S2. Comparison of the incidence of treatment-related adverse events between the combined ICIs group and the single ICI group

Table S3. Comparison of the incidence of treatment-related adverse events in different cycles of immunotherapy

Table S4. Comparison of the incidence of treatment-related adverse events in immunotherapy between the neoadjuvant group and the advanced group

Table S5. List of the incidences of different treatment-related adverse event types in the neoadjuvant ICIs group

Table S6. List of the incidences of different treatment-related adverse event types in the neoadjuvant ICIs plus chemotherapy group

Table S7. List of the incidences of different immune-related adverse event types in the neoadjuvant ICIs group

Table S8. List of the incidences of different immune-related adverse event types in the neoadjuvant ICIs plus chemotherapy group

Table S9. Quality assessment of the included studies

Table S1. Characteristics of the included studies

Study	Cancer type	Treatment group drug	Dosing schedule for immunotherapy	Phase	Number of patients	Criteria for adverse events
Prins (2019)	Glioblastoma	Pembrolizumab	Neoadjuvant stage: Pembrolizumab (200 mg) for 1 cycle;<br>Adjuvant stage: Pembrolizumab (200 mg)	Phase II	16	NA
Nanda (2020)	Breast cancer	Pembrolizumab plus chemotherapy	Pembrolizumab (200 mg) for 4 cycles	Phase II	69	NA
Schumacher (2018)	Melanoma	Ipilimumab plus nivolumab	Neoadjuvant stage: Ipilimumab (3 mg/kg) and nivolumab (1 mg/kg) for 2 cycles;<br>Adjuvant stage: Ipilimumab (3 mg/kg) and nivolumab (1 mg/kg) for 2 cycles	Phase Ib	10	CTCAE v4.03
Sharma (2010)	Bladder cancer	Ipilimumab	Group A: Ipilimumab (3 mg/kg) for 2 cycles;<br>Group B: Ipilimumab (10mg/kg) for 1 cycle or 2 cycles	NA	12	NA
Wargo (2018)	Melanoma	Ipilimumab plus nivolumab	Arm A: Nivolumab (3 mg/kg) for 4 cycles；<br>Arm B: Nivolumab (1 mg/kg) and ipilimumab (3 mg/kg) for 3 cycles	Phase II	Arm A: 11;<br>Arm B: 12	CTCAE v4.0
Chalabi (2020)	Colon cancer	Ipilimumab plus nivolumab	Nivolumab (3 mg/kg) for 2 cycles and ipilimumab (1 mg/kg) for 1 cycle	Phase II	40	CTCAE v4.03
Ferrarotto (2020)	Oropharynx cancer	Durvalumab plus tremelimumab	Group A: Durvalumab (1500mg) and tremelimumab (75 mg) for 2 cycles;	Phase I	Group A: 15;<br>Group B: 14	CTCAE v4.03

			Group B: Durvalumab (1500 mg) for 2 cycles			
Pardoll (2018)	NSCLC	Nivolumab	Nivolumab (3 mg/kg) for 2 cycles	Phase II	22	NA
He (2020)	NSCLC	Sintilimab	Sintilimab (200 mg) for 2 cycles	Phase I	40	CTCAE v4.03
Mitchell (2019)	Melanoma	Pembrolizumab	Pembrolizumab (200 mg) for 1 cycle	Phase I	29	CTCAE v4.0
Loibl (2019)	Breast cancer	Durvalumab plus chemotherapy	Durvalumab (750 mg) for 1 cycle and durvalumab (1500 mg) for 7 cycles	Phase II	88;	CTCAE v4.0
Necchi (2019)	Bladder cancer	Pembrolizumab	Pembrolizumab (200mg) for 3 cycles	Phase II	114	CTCAE v5.0
Powles (2019)	Bladder cancer	Atezolizumab	Atezolizumab (1200 mg) for 2 cycles	Phase II	95	CTCAE v4.03
Blank (2019)	Melanoma	Ipilimumab plus nivolumab	Group A: Ipilimumab (3 mg/kg) and nivolumab (1 mg/kg) for 2 cycles;<br>Group B: Ipilimumab (1 mg/kg) and nivolumab (3mg/kg) for 2 cycles;<br>Group C: Ipilimumab (3 mg/kg) for 2 cycles followed by nivolumab (3 mg/kg) for 2 cycles	Phase II	Group A: 30;<br>Group B: 32;<br>Group C: 27	CTCAE v4.03
Melero (2019)	Glioblastoma	Nivolumab	Neoadjuvant stage: Nivolumab (3 mg/kg) for 1 cycle;<br>Adjuvant stage: Nivolumab (3 mg/kg)	Phase II	30	Common Toxicity Criteria v4.0
Shaughnessy (2020)	Triple-negative breast cancer	Pembrolizumab plus chemotherapy	Pembrolizumab (200 mg) for 4 cycles	Phase III	784	CTCAE v4.0
Schmid (2020)	Triple-negative breast cancer	Pembrolizumab plus chemotherapy	Pembrolizumab (200 mg) for 9 cycles	Phase Ib	60	CTCAE v4.0

Rizvi (2020)	NSCLC	Atezolizumab plus chemotherapy	Atezolizumab (1200 mg) for 4 cycles	Phase II	30	CTCAE v4.0
Tarhini (2014)	Melanoma	Ipilimumab	Ipilimumab (10 mg/kg) for 2 cycles	NA	35	CTCAE v3.0
Topalian (2020)	Merkel cell carcinoma	Nivolumab	Nivolumab (240 mg) for 2 cycles	Phase I/II	39	CTCAE v4.0
Weinhold (2017)	NSCLC	Ipilimumab plus chemotherapy	Ipilimumab (10 mg/kg) for 2 cycles	Phase II	24	NA
Daud (2020)	Melanoma	Nivolumab; Ipilimumab	NA	NA	17	CTCAE v4.0
Mittendorf (2020)	Triple-negative breast cancer	Atezolizumab plus chemotherapy	Atezolizumab (840 mg) for 6 cycles	Phase III	333	CTCAE v4.0
Provencio (2020)	NSCLC	Nivolumab plus chemotherapy	Nivolumab (360 mg) for 3 cycles	Phase II	46	CTCAE v4.0
Forde (2020)	NSCLC	Nivolumab plus ipilimumab	Nivolumab (3 mg/kg) for 3 cycles and ipilimumab (1 mg/kg) for 1 cycle	Phase II	9	CTCAE v4.1
Schoenfeld (2020)	Oral cavity squamous cell carcinoma	Nivolumab; Ipilimumab	Group A: Nivolumab (3 mg/kg) for 2 cycles;<br>Group B: Nivolumab (3 mg/kg) for 2 cycles and ipilimumab (1 mg/kg) for 1 cycle	Phase II	30	CTCAE v4.0
Uppaluri (2020)	Head and neck cancer	Pembrolizumab	Pembrolizumab (200 mg) for 1 cycle	Phase II	36	CTCAE v4.0
Gao (2020)	Bladder cancer	Durvalumab plus Tremelimumab	Durvalumab (1500 mg/kg) and tremelimumab (75 mg/kg) for 2 cycles	Phase I	28	CTCAE v4.03

Abbreviations: CTCAE: Common Terminology Criteria for Adverse Events; NA: not applicable; NSCLC: non-small cell lung cancer

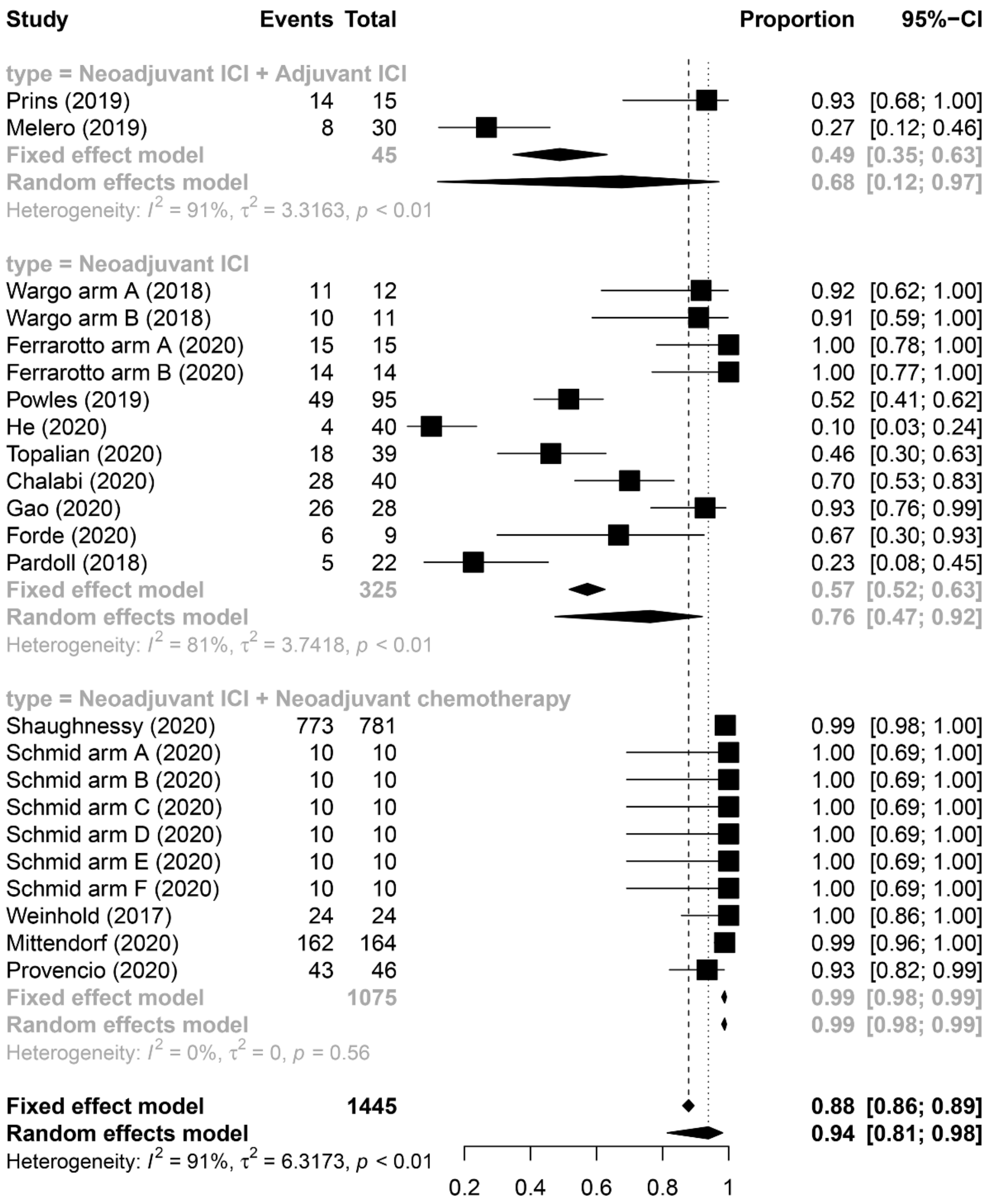

Figure S1. Forest plots of the incidence of all-grade treatment-related adverse events. ICI: immune checkpoint inhibitor; CI: confidence interval.

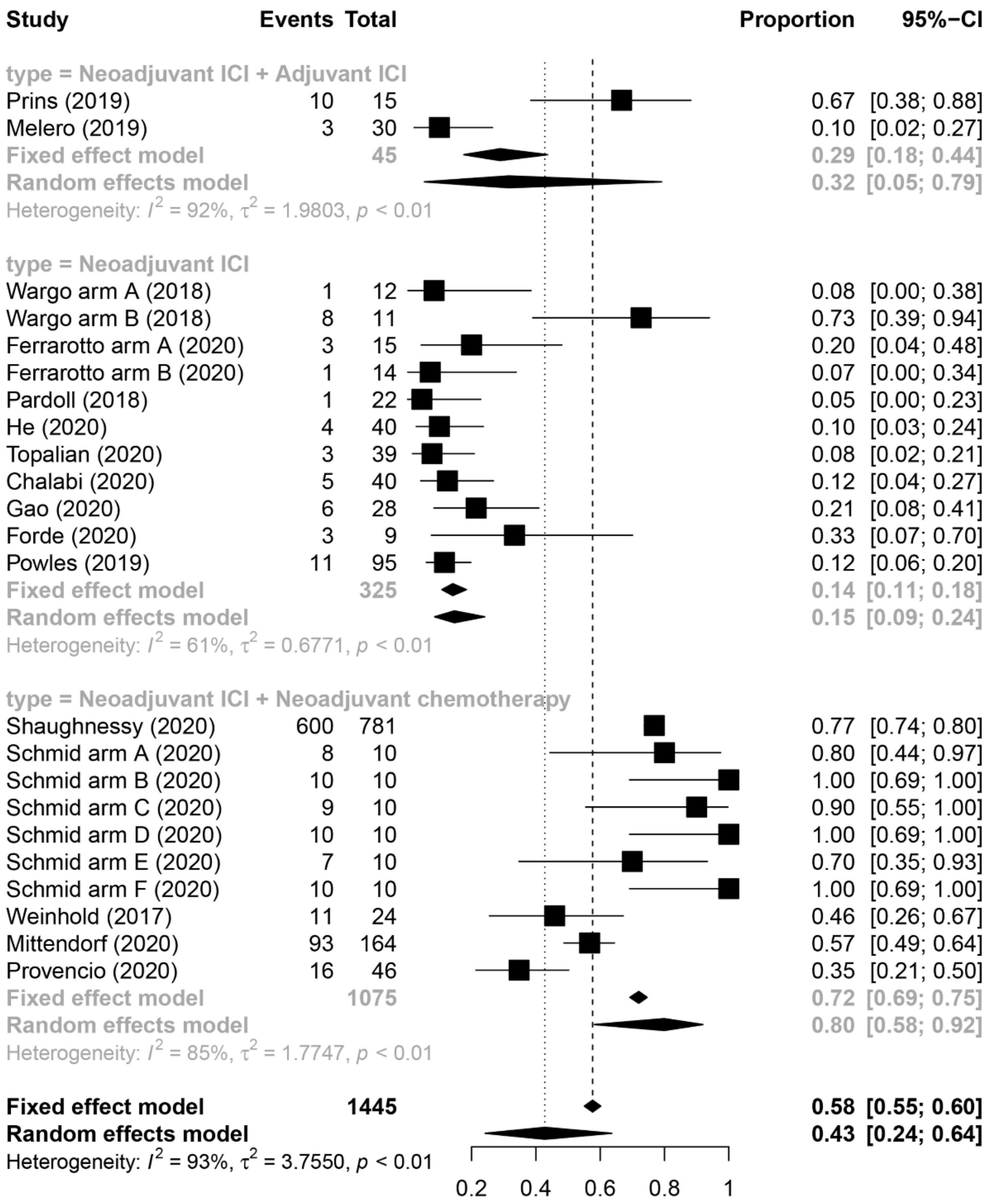

Figure S2. Forest plots of the incidence of high-grade treatment-related adverse events. ICI: immune checkpoint inhibitor; CI: confidence interval.

Table S2. Comparison of the incidence of treatment-related adverse events between the combined ICIs group and the single ICI group

Type	Treatment group	Proportion (95% CI) (%)	*P*
All-grade trAEs	Single ICI	60 (38 - 82)	/
	Combined ICIs	84 (73 - 94)	0.057
High-grade trAEs	Single ICI	10 (7 - 15)	/
	Combined ICIs	24 (10 - 48)	0.148

Abbreviations: trAEs: treatment-related adverse events; CI: confidence interval; ICIs: immune checkpoint inhibitors.

Table S3. Comparison of the incidence of treatment-related adverse events in different cycles of immunotherapy

Type	Treatment group	Cycles	Proportion (95% CI) (%)	*P*
All-grade trAEs	Neoadjuvant ICIs	< 3	61 (33 - 89)	/
		≥ 3	81 (69 - 94)	0.003
	Neoadjuvant ICIs plus chemotherapy	< 4	98 (95 - 99)	/
		≥ 4	99 (98 - 100)	0.242
High-grade trAEs	Neoadjuvant ICIs	< 3	11 (8 - 16)	/
		≥ 3	26 (8 - 57)	0.295
	Neoadjuvant ICIs plus chemotherapy	< 4	47 (33 - 61)	/
		≥ 4	90 (80 - 99)	< 0.001

Abbreviations: trAEs: treatment-related adverse events; CI: confidence interval; ICIs: immune checkpoint inhibitors.

Table S4. Comparison of the incidence of treatment-related adverse events in immunotherapy between the neoadjuvant group and the advanced group

Type	Treatment group	Stage	Proportion (95% CI) (%)	*P*
All-grade trAEs	ICIs	Advanced (1)	71 (68 - 74)	/
		Early (Neoadjuvant)	76 (47 - 92)	0.696
	ICIs plus chemotherapy	Advanced (2)	98 (97 - 99)	/
		Early (Neoadjuvant)	99 (98 - 99)	0.227
High-grade trAEs	ICIs	Advanced (1)	15 (13 - 16)	/
		Early (Neoadjuvant)	15 (9 - 24)	0.530
	ICIs plus chemotherapy	Advanced (2)	67 (62 - 72)	/
		Early (Neoadjuvant)	80 (58 - 92)	0.222

Abbreviations: trAEs: treatment-related adverse events; CI: confidence interval; ICIs: immune checkpoint inhibitors.

Table S5. List of the incidences of different treatment-related adverse event types in the neoadjuvant ICIs group

Event	Incidence of all grade (95% CI)	Incidence of high grade (95% CI)
Fatigue	0.25 (0.15-0.38)	NA
Transaminitis	0.23 (0.10-0.44)	0.03 (0.00-0.07)
Rash	0.17 (0.08-0.35)	NA
Hyperthyroidism	0.15 (0.12-0.20)	NA
Anemia	0.14 (0.07-0.26)	NA
Hypomagnesemia	0.14 (0.07-0.26)	NA
Pruritus	0.11 (0.06-0.20)	NA
Arthralgia	0.11 (0.05-0.24)	NA
Lipase increased	0.10 (0.06-0.17)	0.05 (0.02-0.10)
Diarrhea	0.10 (0.05-0.18)	NA
Headache	0.10 (0.04-0.23)	NA
Hyponatremia	0.08 (0.04-0.15)	NA
Anorexia	0.08 (0.04-0.14)	NA
Vomiting	0.08 (0.03-0.22)	NA
Amylase increased	0.08 (0.03-0.20)	NA
Cough	0.08 (0.02-0.25)	NA
Nausea	0.07 (0.05-0.11)	NA
Hypothyroidism	0.07 (0.05-0.10)	NA
Dry skin	0.07 (0.03-0.17)	NA
AST increased	0.07 (0.02-0.26)	0.01 (0.00-0.04)
ALT increased	0.07 (0.02-0.26)	0.01 (0.00-0.04)
Fever	0.06 (0.04-0.10)	NA
Myalgia	0.06 (0.03-0.11)	NA
Abdominal pain	0.06 (0.02-0.22)	0.02 (0.00-0.21)
Bilirubin increased	0.06 (0.02-0.15)	0.01 (0.00-0.07)
Autoimmune disorders	0.05 (0.02-0.15)	0.01 (0.00-0.07)
Infusion−related reaction	0.05 (0.02-0.12)	NA
Back pain	0.05 (0.02-0.12)	NA
Dry mouth	0.05 (0.02-0.09)	NA
Asthenia	0.05 (0.01-0.17)	NA
Colitis	0.04 (0.02-0.11)	0.03 (0.00-0.07)
Sinus tachycardia	0.04 (0.02-0.11)	NA
Creatinine increased	0.04 (0.02-0.09)	NA
Constipation	0.04 (0.02-0.09)	NA
Pneumonitis	0.04 (0.02-0.08)	0.02 (0.00-0.07)

Alkaline phosphatase increased	0.04 (0.02-0.07)	NA
Dyspnea	0.04 (0.01-0.16)	NA
Psoriasis	0.04 (0.01-0.15)	NA
Hypokalemia	0.04 (0.01-0.14)	NA
dizziness	0.04 (0.01-0.13)	NA
Edema limbs	0.04 (0.01-0.13)	NA
Flu−like symptoms	0.04 (0.01-0.13)	NA
Adrenal insufficiency	0.03 (0.01-0.11)	NA
Vision blurred	0.03 (0.01-0.11)	NA
Arthritis	0.03 (0.01-0.07)	NA
Creatine phosphokinase increased	0.02 (0.01-0.07)	NA
Hypertension	0.02 (0.01-0.07)	NA
Hyperglycemia	0.02 (0.01-0.06)	NA
Lymphocyte count increased	0.02 (0.00-0.06)	NA
Pain	0.02 (0.00-0.06)	NA
Lung infection	0.01 (0.00-0.06)	NA

Abbreviations: CI: confidence interval; ICIs: immune checkpoint inhibitors; ALT: alanine transaminase; AST: aspartate aminotransferase. NA: not applicable.

Table S6. List of the incidences of different treatment-related adverse event types in the neoadjuvant ICIs plus chemotherapy group

Event	Incidence of all grade (95% CI)	Incidence of high grade (95% CI)
Neutropenia	0.71 (0.53-0.89）	0.53 (0.31-0.74)
Nausea	0.62 (0.52-0.72）	0.01 (0.00-0.02)
Alopecia	0.59 (0.48-0.70)	0.01 (0.00-0.01)
Anemia	0.53 (0.38-0.68）	0.08 (0.03-0.15)
Fatigue	0.53 (0.41-0.65）	0.02 (0.01-0.03)
Headache	0.38 (0.23-0.53)	NA
Diarrhea	0.36 (0.25-0.47)	0.02 (0.01-0.03)
ALT increased	0.32 (0.16-0.48)	0.03 (0.02-0.04)
Peripheral sensory neuropathy	0.32 (0.17-0.47）	0.03 (0.00-0.06)
Myalgia	0.31 (0.24-0.39)	0.01 (0.00-0.04)
Insomnia	0.31 (0.14-0.48)	NA
Constipation	0.30 (0.20-0.40)	NA
Arthralgia	0.29 (0.17-0.41)	NA
Thrombocytopenia	0.28 (0.12-0.44）	0.02 (0.00-0.05)
AST increased	0.27 (0.06-0.48)	0.04 (0.02-0.07)
Cough	0.27 (0.19-0.34)	NA
Vomiting	0.26 (0.19-0.32）	NA
Bone pain	0.25 (0.12-0.38)	NA
Hot flush	0.24 (0.13-0.35)	NA
Rash	0.22 (0.20-0.25）	0.01 (0.00-0.01)
Stomatitis	0.22 (0.14-0.29)	0.01 (0.01-0.02)
Asthenia	0.22 (0.12-0.31)	0.03 (0.02-0.04)
Appetite decreased	0.21 (0.11-0.31)	0.01 (0.00-0.01)
Mucositis	0.21 (0.07-0.34)	0.01 (0.00-0.03)
Dyspnea	0.20 (0.09-0.32)	NA
Anorexia	0.19 (0.11-0.27)	0.01 (0.00-0.03)
Dysgeusia	0.19 (0.09-0.28)	NA
Epistaxis	0.18 (0.12-0.25)	NA
Fever	0.17 (0.15-0.20)	0.01 (0.00-0.01)
Neuropathy peripheral	0.16 (0.06-0.25)	NA

Pruritus	0.16 (0.11-0.21)	NA
Vertigo	0.15 (0.00-0.36)	NA
Abdominal pain	0.15 (0.00-0.30)	NA
Dry Skin	0.15 (0.06-0.24)	NA
Oedema peripheral	0.15 (0.10-0.20)	NA
Depression	0.12 (0.02-0.21)	NA
Sleep disturbance	0.12 (0.00-0.34)	NA
Lacrimation increased	0.12 (0.09-0.16)	NA
Pain	0.12 (0.04-0.20)	NA
Infusion-related reaction	0.12 (0.08-0.16)	NA
Upper respiratory tract infection	0.12 (0.07-0.16)	NA
Oedema	0.11 (0.00-0.31)	NA
Dyspepsia	0.11 (0.07-0.15)	NA
Febrile neutropenia	0.10 (0.05-0.15）	NA
Back pain	0.10 (0.05-0.16)	NA
Weight decreased	0.10 (0.04-0.16)	NA
Paresthesia	0.09 (0.01-0.17)	NA
Hypertension	0.09 (0.05-0.12)	NA
Hypotension	0.06 (0.00-0.13)	NA
Dry eye	0.06 (0.02-0.09)	NA
Creatinine increased	0.05 (0.00-0.12）	0.01 (0.00-0.09)
Hypomagnesemia	0.04 (0.00-0.13)	NA
Hypophosphatemia	0.04 (0.00-0.13)	NA
Bilirubin increased	0.03 (0.00-0.07）	NA
Hyponatremia	0.02 (0.00-0.05)	NA

Abbreviations: CI: confidence interval; ICIs: immune checkpoint inhibitors; ALT: alanine transaminase; AST: aspartate aminotransferase. NA: not applicable.

Table S7. List of the incidences of different immune-related adverse event types in the neoadjuvant ICIs group

Event	Incidence of all grade (95% CI)	Incidence of high grade (95% CI)
Rash	0.29 (0.16-0.46)	NA
Transaminitis	0.20 (0.10-0.36)	0.03 (0.01-0.08)
AST increased	0.19 (0.14-0.26)	0.02 (0.00-0.05)
Pruritus	0.17 (0.11-0.26)	NA
ALT increased	0.17 (0.08-0.33)	0.04 (0.00-0.11)
Diarrhea	0.15 (0.09-0.26)	NA
Hyperthyroidism	0.15 (0.09-0.24)	NA
Anemia	0.11 (0.06-0.18)	NA
Amylase increased	0.10 (0.07-0.15)	0.02 (0.00-0.05)
Arthralgia	0.10 (0.07-0.15)	NA
Lipase increased	0.10 (0.06-0.15)	0.03 (0.01-0.07)
Gamma−glutamyltransferase increased	0.10 (0.05-0.16)	0.04 (0.01-0.08)
Fever	0.09 (0.07-0.13)	NA
Hypothyroidism	0.08 (0.06-0.11)	NA
Abdominal pain	0.07 (0.04-0.12)	NA
Colitis	0.06 (0.03-0.15)	0.04 (0.01-0.08)
Flu−like symptoms	0.06 (0.03-0.12)	NA
Hyponatremia	0.06 (0.03-0.10)	0.02 (0.00-0.06)
Uveitis	0.06 (0.01-0.2)	NA
Adrenal insufficiency	0.05 (0.02-0.09)	0.01 (0.00-0.04)
Pneumonitis	0.04 (0.02-0.08)	0.02 (0.00-0.07)
Alkaline phosphatase increased	0.04 (0.02-0.07)	NA
Bilirubin increased	0.03 (0.01-0.10)	0.01 (0.00-0.03)
Hyperglycemia	0.02 (0.01-0.06)	NA

Abbreviations: CI: confidence interval; ICIs: immune checkpoint inhibitors; ALT: alanine transaminase; AST: aspartate aminotransferase. NA: not applicable.

Table S8. List of the incidences of different immune-related adverse event types in the neoadjuvant ICIs plus chemotherapy group

Event	Incidence of all grade (95% CI)	Incidence of high grade (95% CI)
Anemia	0.37 (0.14-0.59)	0.16 (0.14-0.18)
Diarrhea	0.32 (0.24-0.40)	0.02 (0.01-0.03)
ALT increased	0.25 (0.22-0.28)	0.05 (0.04-0.07)
Pruritus	0.20 (0.10-0.30)	NA
AST increased	0.20 (0.14-0.25)	0.05 (0.02-0.09)
Arthralgia	0.19 (0.09-0.29)	NA
Fever	0.18 (0.15-0.20)	0.01 (0.01-0.02)
Rash	0.11 (0.03-0.19)	0.01 (0.01-0.02)
Hypothyroidism	0.08 (0.04-0.11)	0.01 (0.00-0.04)
Hyperthyroidism	0.06 (0.03-0.09)	NA
Pneumonitis	0.02 (0.00-0.04)	NA
Colitis	0.02 (0.00-0.04)	NA

Abbreviations: CI: confidence interval; ICIs: immune checkpoint inhibitors; ALT: alanine transaminase; AST: aspartate aminotransferase. NA: not applicable.

Table S9. Quality assessment of the included studies

	Sequence generation	Allocation concealment	blinding	Incomplete outcome data	selective outcome reporting	other sources of bias
Wargo et al. 2019	+	+	-	+	+	+
Schumacher et al. 2018	+	+	-	+	+	+
Nanda et al. 2020	+	+	-	+	+	+
Sharma et al. 2010	-	-	-	+	+	+
Prins et al. 2019	+	+	-	+	+	+
Chalabi et al. 2020	+	-	-	+	+	+
Ferrarotto et al. 2020	+	+	-	+	+	+
Pardoll et al. 2018	-	NA	-	+	+	+
Gao et al. 2020	-	NA	-	+	+	-
He et al. 2020	-	NA	-	+	+	+
Mitchell et al. 2019	-	NA	-	+	+	+
Daud et al. 2020	-	NA	-	+	+	-
Loibl et al. 2019	+	+	+	+	+	+
Mittendorf et al. 2020	+	+	+	+	+	+
Necchi et al. 2020	-	NA	-	+	+	+
Powles et al. 2019	-	NA	-	+	+	+
Provencio et al. 2020	-	NA	-	+	+	+

Forde et al. 2020	-	NA	-	+	+	-
Blank et al. 2019	+	-	-	+	+	+
Melero et al. 2019	-	NA	-	+	+	+
Shaughnessy et al. 2020	+	+	+	+	+	+
Schmid et al. 2020	+	NA	-	+	+	+
Schoenfeld et al. 2020	+	NA	-	+	+	+
Rizvi et al. 2020	-	NA	-	+	+	+
Tarhini et al. 2014	-	NA	-	+	+	+
Topalian et al. 2020	-	NA	-	+	+	+
Uppaluri et al. 2020	-	-	-	+	+	-
Weinhold et al. 2017	-	-	-	+	+	+

Quality assessment: +: low risk of bias; -: high risk of bias; NA: not applicable.

References

1. Wang Y, Zhou S, Yang F, Qi X, Wang X, Guan X, et al. Treatment-Related Adverse Events of PD-1 and PD-L1 Inhibitors in Clinical Trials: A Systematic Review and Meta-analysis. *JAMA oncology* (2019) 5(7):1008-19. doi: 10.1001/jamaoncol.2019.0393
2. Zhou X, Yao Z, Bai H, Duan J, Wang Z, Wang X, et al. Treatment-related adverse events of PD-1 and PD-L1 inhibitor-based combination therapies in clinical trials: a systematic review and meta-analysis. *The Lancet Oncology* (2021) 22(9):1265-74. doi: 10.1016/s1470-2045(21)00333-8
